# Matured Hop Bittering Components Induce Thermogenesis in Brown Adipose Tissue *via* Sympathetic Nerve Activity

**DOI:** 10.1371/journal.pone.0131042

**Published:** 2015-06-22

**Authors:** Yumie Morimoto-Kobayashi, Kazuaki Ohara, Chika Takahashi, Sayoko Kitao, Guanying Wang, Yoshimasa Taniguchi, Mikio Katayama, Katsuya Nagai

**Affiliations:** 1 Research Laboratories for Health Science & Food Technologies, KIRIN Company, Ltd., Yokohama, Kanagawa, Japan; 2 ANBAS Corporation, Osaka, Japan; The Ohio State University, UNITED STATES

## Abstract

Obesity is the principal symptom of metabolic syndrome, which refers to a group of risk factors that increase the likelihood of atherosclerosis. In recent decades there has been a sharp rise in the incidence of obesity throughout the developed world. Iso-α-acids, the bitter compounds derived from hops in beer, have been shown to prevent diet-induced obesity by increasing lipid oxidation in the liver and inhibition of lipid absorption from the intestine. Whereas the sharp bitterness induced by effective dose of iso-α-acids precludes their acceptance as a nutrient, matured hop bittering components (MHB) appear to be more agreeable. Therefore, we tested MHB for an effect on ameliorating diet-induced body fat accumulation in rodents. MHB ingestion had a beneficial effect but, compared to iso-α-acids and despite containing structurally similar compounds, acted *via* different mechanisms to reduce body fat accumulation. MHB supplementation significantly reduced body weight gain, epididymal white adipose tissue weight, and plasma non-esterified free fatty acid levels in diet-induced obese mice. We also found that uncoupling protein 1 (UCP1) expression in brown adipose tissue (BAT) was significantly increased in MHB-fed mice at both the mRNA and protein levels. In addition, MHB administration in rats induced the β-adrenergic signaling cascade, which is related to cAMP accumulation in BAT, suggesting that MHB could modulate sympathetic nerve activity innervating BAT (BAT-SNA). Indeed, single oral administration of MHB elevated BAT-SNA in rats, and this elevation was dissipated by subdiaphragmatic vagotomy. Single oral administration of MHB maintained BAT temperature at a significantly higher level than in control rats. Taken together, these findings indicate that MHB ameliorates diet-induced body fat accumulation, at least partly, by enhancing thermogenesis in BAT *via* BAT-SNA activation. Our data suggests that MHB is a useful tool for developing functional foods or beverages to counteract the accumulation of body fat.

## Introduction

Metabolic syndrome, which is closely linked to atherosclerosis, is now recognized as a major worldwide public health problem [1]. Obesity is associated with insulin resistance, hyperlipidemia and hypertension, and is a major symptom of metabolic syndrome [2]. Because excess energy intake is a key cause of obesity, appropriate dietary modification and increased energy expenditure are obvious therapeutic approaches [3].

BAT is a major organ for cold- and diet-induced adaptive thermogenesis [4,5]. Uncoupling proteins (UCPs) can uncouple respiration from ATP synthesis by short-circuiting the inward flow of protons across the inner mitochondrial membrane. Although UCP1, UCP2 and UCP3 are all expressed in BAT, UCP1 is thought to be the key regulator of adaptive thermogenesis in this tissue [6]. Thus UCP1 contributes to maintaining body weight by helping to control energy expenditure. BAT has long been recognized to be abundant in small rodents but absent or negligible in adult humans. However, recent studies, using fluorodeoxyglucose (FDG)-PET in combination with computed tomography (CT) [7], revealed that adult humans have metabolically active BAT. Since the publication of these findings, considerable effort has been devoted to understanding the regulation of BAT activity with the aim of managing obesity and related metabolic disorders.

Hops, the immature inflorescences of the female hop plant (*Humulus lupulus* L.), have been widely used for beer production to add flavor and bitterness. Iso-α-acids, major bitter components in beer, are converted from α-acids in hops by isomerization during the brewing process. Several studies have established the many health benefits of ingesting iso-α-acids [8–10]. In terms of prevention of obesity, isomerized hop extract, which consists primarily of iso-α-acids, was shown to prevent diet-induced obesity by the modulation of lipid oxidation in the liver *via* PPARα activation and inhibition of intestinal lipid absorption [11]. However, it is difficult to add an effective dose of iso-α-acids to foods because of their bitterness. It is now well established that the α- and β-acid content of hops rapidly decreases during storage, while other bittering components accumulate [12]. Although these components were thought to be derived from α- and β-acids, there is little published information concerning their identity. Recently, however, we revealed that these bittering components primarily consist of α-acid oxides, which possess a common β-tricarbonyl moiety in their structures similar to α-, β- and iso-α-acids [13–15]. A recent study indicated that the oxidation products of α-acids may result in a more agreeable bitterness than iso-α-acids [16], suggesting that these bittering components may be more useful for food applications.

The physiological effects of bittering components in oxidized hops have not been reported. Here, we prepared bittering components from oxidized hops, namely matured hop bittering components (MHB), and then evaluated the effects of MHB on body fat accumulation. Furthermore, the underlying mechanism was investigated.

## Materials and Methods

### Materials and chemicals

Hop pellets and isomerized hop extract (as standards for iso-α-acids) were purchased from HopSteiner (Mainburg, Germany). Tricyclooxyisohumulones A and B were prepared as described [14]. As standards of α- and β-acids, ICE2 was purchased from American Society of Brewing Chemists (St. Paul, MN).

### Preparation and HPLC analysis of MHB

MHB was prepared as described previously from hop pellets [15]. MHB was analyzed and quantified by HPLC using a previously reported method [14,15].

High resolution mass spectra of the components of MHB were measured using a Thermo Scientific LTQ Orbitrap mass spectrometer (Thermo Fisher Scientific, San Jose, CA).

### Animals

Male C57BL/6J mice and male Wistar rats were purchased from Charles River Japan (Kanagawa, Japan) or Kiwa Laboratory Animals Co. Ltd. (Wakayama, Japan). High fat diet (HFD)-feeding study was with obesity-prone C57BL/6J mice, other single oral administration studies were with Wistar rats due to technical difficulties using mice. Animals were maintained under a constant 12-h light/dark cycle (light from 8:00 a.m. to 8:00 p.m.). Mice were acclimatized by feeding AIN93G (Research Diets, Inc., New Brunswick, NJ), and rats were acclimatized by feeding CE-2 (Clea Japan, Tokyo, Japan) or type MF (Oriental Yeast Co., Tokyo, Japan), for at least five days. In the acclimatization period, the animals were allowed free access to water and food, and were then used for each experiment. The animal care and handling procedures of the study for recording BAT-SNA were approved by the Institutional Animal Care and Use Committee of the ANBAS Corporation in accordance with the Guidelines for Animal Experiments Issued by the Science Council of Japan on June 1, 2006 (Permit Number: 317 and 345). Other studies were conducted according to the Guidelines for Ethical Animal Care, Handling, and Termination from Kirin Company, which are in line with International and Japanese Guidelines for Animal Care and Welfare, and were approved by the Institutional Animal Care and Use Committee of the Kirin Company (Permit Number: YO13-00026, YO13-00058 and YO14-00085). All surgery was performed under anesthesia, and all efforts were made to minimize suffering.

### Administration of a MHB-supplemented HFD to mice

Six-week-old male C57BL/6J mice were divided into two groups (n = 12 mice/group) that were matched for body weight. Mice were allowed free access to water, and fed a ‘western’-type HFD containing 21% fat by weight; i.e., 42% of calorie intake as fat (TD88137, Harlan Teklad, Madison, WI) [17–20], with MHB for 9 wk. Mice fed a HFD containing MHB were administered increasing amounts of MHB up to 0.2% in stages i.e., 0.025, 0.05 and 0.1% for 1 wk, respectively, and then 0.2% for the remaining period. For the control group, mice were fed a HFD with the vehicle of MHB for 9 wk. Since our preliminary study in mice indicated that MHB supplementation in the diet tended to slightly reduce food intake, feeding amount of control group was slightly adjusted to prevent the divergence of food intake between the two groups during the experimental period. The calorie content of the HFD with or without 0.2% MHB supplementation were both 19.7 kJ/g. Energy values for each diet were calculated from the macronutrient composition using values of 17, 17 and 38 kJ/g for carbohydrate, protein and lipid, respectively. Mice were individually housed in cages. Food intake was measured every 1–3 days during the course of the study using Roden CAFE food dispensers (Oriental Yeast Co., Tokyo, Japan) to minimize dispersion of the diet. Body weight was measured twice/wk during the course of the study. On the final day of the experiment, blood was collected by orbital sinus puncture under diethyl ether anesthesia, and mice were sacrificed by exsanguination from the orbital sinus and cervical dislocation under diethyl ether anesthesia. Tissues were dissected from each mouse and immediately frozen in liquid nitrogen.

### Plasma analysis in mice fed a HFD

The levels of plasma triacylglycerol (TG), total cholesterol, non-esterified fatty acid (NEFA) and glucose were measured using the Triglyceride G Test Wako, Cholesterol E Test Wako, NEFA C Test Wako, and Glucose C Test Wako (Wako Pure Chemicals, Osaka, Japan) according to the manufacturer’s instructions.

### Measurement of fecal lipid content in mice fed a HFD

Feces from the cages in which mice were individually housed were collected twice during the eighth wk of the experiment. After freeze-drying, the total lipid content of the feces was extracted in chloroform:methanol (2:1, v/v), as described previously [21]. The amount of extracted lipid fraction was measured gravimetrically, followed by subtraction for the amount of MHB in that fraction. The amount of MHB was measured by the method described above.

### Quantitative real-time PCR in tissues of mice fed a HFD

Total RNA was extracted from mouse tissues with Isogen (Nippon Gene, Toyama, Japan), and purified using RNeasy (Qiagen, Hilden, Germany) according to the manufacturer’s instructions. cDNA was synthesized from total RNA by reverse transcription using ThermoScript RT-PCR System (Invitrogen, Carlsbad, CA). Quantitative real-time PCR was performed with a LightCycler 480 instrument (Roche Diagnostics, Tokyo, Japan) using SYBR Premix Ex Taq (Takara Bio, Shiga, Japan). Levels of mRNA were normalized to that of *glyceraldehyde -3-phosphate dehydrogenase* (*GAPDH*) mRNA. Primer sequences for *UCP1*, *peroxisome proliferator-activated receptor gamma coactivator-1α* (*PGC-1α*), *carnitine palmitoyltransferase 1β (CPT1β*), *acyl-CoA oxidase* (*ACO*), *PR domain containing 16* (*PRDM16*), *peroxisome proliferator-activated receptor γ* (*PPARγ*), *liver X receptor α* (*LXRα*), *sterol regulatory element-binding protein-1c* (*SREBP-1c*), *acetyl-CoA carboxylase 1* (*ACC1*), *fatty acid synthase* (*FAS*), *medium-chain acyl-CoA dehydrogenase* (*MCAD*), *UCP3* and *GAPDH* are provided in S1 Table.

### Immuno-blot analysis of UCP1 in BAT of mice fed a HFD

The mitochondrial fraction was prepared using Mitochondria Isolation Kit (Biochain, Newark, CA) according to the manufacturer’s instructions. The mitochondrial protein content was measured with a DC protein assay kit (Bio-Rad, Hercules, CA). Bovine serum albumin was used for generating a standard curve. The mitochondrial fraction (2.5 μg) isolated from BAT in each mouse were separated by SDS-PAGE. Anti-UCP1 rabbit polyclonal antibody (1:2000, Calbiochem, Darmstadt, Germany) and anti-Ubiquinol-cytochrome C reductase core protein 1 (UQCRC1) rabbit polyclonal antibody (1:2000, Abcam Plc., Cambridge, UK) were used as primary antibodies and Anti-rabbit IgG, HRP-linked whole antibody (1:20000, GE Healthcare Life Science, Amersham, UK) as a secondary antibody. Antibody reactivity was detected by ECL Prime Western Blotting Detection Reagent (GE Healthcare Life Science). The density of the UCP1 protein band was quantified by densitometric and image analysis, and normalized to that of the UQCRC1 band as internal standard by use of LAS-4000 (Fujifilm, Tokyo, Japan). Data were presented as the value relative to the control group.

### Recording of BAT-SNA in rats given a single oral administration of MHB

BAT-SNA was determined as described previously [22,23]. Briefly, after fasting for 3 h, 9-wk-old male Wistar rats were anesthetized with urethane (1 g/kg, i.p.). For oral administration of MHB solution (2 mg/kg or 10 mg/kg), an oral cannula was inserted into the gastric cavity (n = 3 rats/group). After tracheal cannulation and lapatomy, distal ends of sympathetic nerves innervating the interscapular BAT were exposed, ligated and subsequently connected with a pair of silver wire electrodes. The body temperature was maintained at 35.0 ± 0.5°C using a heat pad. After stabilizing for 30–60 min, the electrical signals from the electrodes were collected, amplified, filtered and monitored on an oscilloscope. Nerve activity was analyzed by conversion of the raw data to standard pulses by using a window discriminator. In subgroups of rats, subdiaphragmatic vagotomy was performed before the recording of BAT-SNA. Sham surgery was performed in the same way but without cutting the vagus nerve. MHB was dissolved in 3.6 mM potassium carbonate, and administrated into the gastric cavity of the rats using the oral cannula. For the control group, rats were administered vehicle in which the pH was adjusted to the same value as that of the MHB solution (pH 7) using 1 N HCl.

### Measurement of the cAMP level in BAT of rats given a single oral administration of MHB

After fasting for 3 h, 9-wk-old male Wistar rats were pretreated with the β-adrenergic antagonist propranolol (Sigma-Aldrich, ST. Louis, MO) (10 mg/kg, i.p.) or saline to investigate involvement of the β-adrenergic receptor. Thirty minutes after the pretreatment, rats were orally administered MHB solution (10 mg/kg) *via* a stomach tube (n = 10 rats/group). As a control group, rats were administered vehicle with the pH adjusted to the same value as that of the MHB solution (pH 7) using 1 N HCl. Rats were killed 3 h after administration of MHB by exsanguination from the abdominal aorta under diethyl ether anesthesia, and interscapular BAT was then removed. For the preparation of the lysates of BAT and the measurement of cAMP level in the lysates, we used DetectX High Sensitivity Direct cAMP Chemiluminescent Immunoassay Kit (Arbor Assays, Ann Arbor, MI) following the manufacturer’s instructions. In each case, the cAMP level is presented as the ratio of cAMP to protein.

### Measurement of BAT temperature (T_BAT_) in rats given a single oral administration of MHB

Eight days before the measurement of T_BAT_, a temperature transmitter (TA11TA-F10, Data Sciences International (DSI), St. Paul, MN) was inserted between an interscapular BAT pad and the trapezius muscle, and the incision was sutured with silk thread under inhalation anesthesia of isoflurane (Escain, Mylan Japan, Tokyo, Japan). The output signal was processed using a receiver (RPC-1, DSI), a data exchange matrix, and an ambient pressure reference monitor (APR-1, DSI). The data obtained by this system were analyzed by Dataquest ART 4.0 Acquisition system (DSI). On the experimental day, 9-wk-old male Wistar rats were anesthetized with urethane (1.5 g/kg, i.p.) after fasting for 3 h (n = 4 rats/group). For oral administration of MHB solution (10 mg/kg), an oral cannula was inserted into the gastric cavity. Rats were placed on fixed temperature heating pads (34°C) during the experiments. After the administration of MHB, T_BAT_ was measured for a 3 h period. As a control group, rats were administered vehicle with the pH adjusted to the same value as that of the MHB solution (pH 7) using 1 N HCl.

### Statistical analysis

All values are means ± SEM. Statistical differences were analyzed by the appropriate statistical methods specified in the figure legends below. *P* values < 0.05 were considered statistically significant. Statistical analysis was performed by using KaleidaGraph (Synergy Software, Reading, PA).

## Results

### HPLC analysis of MHB

MHB was subjected to HPLC analysis and the eluate monitored at 270 nm (S1A Fig). Our results confirmed that MHB comprises only trace amounts of α-acids, β-acids and iso-α-acids (S1B and S1C Fig). MHB was recently found to be primarily composed of oxidative α-acid derivatives [13–15]. Actually, from direct comparison of HPLC retention times and high resolution mass spectra with authentic standards, MHB was confirmed to contain tricyclooxyisohumulones A and B, as oxides of α-acids (S1D and S1E and S2 Figs).

### MHB ameliorated HFD-induced body fat accumulation in mice

**Fig 1:**
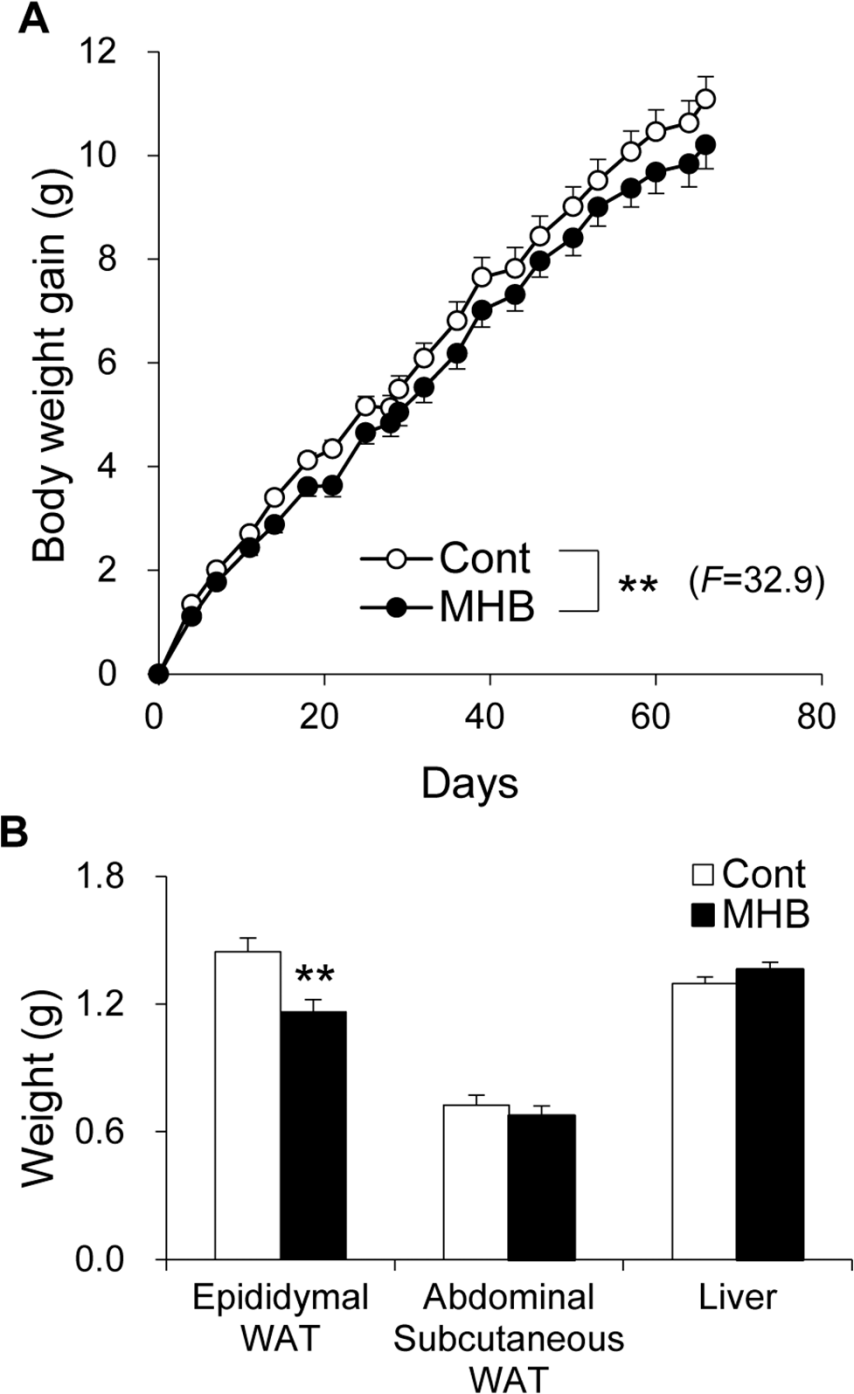
MHB ameliorated HFD-induced body fat accumulation in mice. (A) Body weight gain of HFD-fed mice with or without MHB supplementation. Data are presented as means ± SEM. n = 12 mice/group. **P < 0.01 (by analysis of variance (ANOVA) with repeated measures). (B) Epididymal WAT, abdominal subcutaneous WAT and liver weight. Data are presented as means ± SEM. n = 12 mice/group. **P < 0.01 (by unpaired Student’s *t*-test).

The body weight of mice fed HFD with and without MHB supplementation was monitored. As shown in Fig 1A, weight gain was significantly suppressed in the group fed a diet containing MHB compared to mice fed HFD without supplement (*P* < 0.01). There was no significant difference in food intake between the two groups (Table 1 and S3A Fig). Moreover, there was no significant correlation between total food intake and body weight gain (S3B Fig, Pearson correlation coefficient *r* = 0.21, *p* = 0.32). Epididymal white adipose tissue (WAT) weight of MHB-fed mice was also significantly decreased to 80.4% that of the control mice (Fig 1B, *P* < 0.01). There were no significant differences in plasma TG, total cholesterol and glucose levels between the two groups. However, the plasma NEFA level in mice fed HFD containing MHB was suppressed by 21.7% (*P* < 0.01) compared to that of the control group (Table 2). There was no significant difference in fecal excretion of lipids between the two groups (Table 1), suggesting that MHB does not affect the absorption rate of dietary fat under our experimental conditions.

**Table 1:** Total food intake of mice fed on HFD with or without MHB supplementation and the effect of MHB on fecal lipid content.

	Cont			MHB		
fecal lipids excretion (mg/7 days)	38.63	±	0.91	39.61	±	2.23
total food intake (g/66 days)	172.40	±	0.84	169.32	±	1.52

Data are presented as means ± SEM. n = 12 mice/group. No significant differences were observed by unpaired Student’s *t*-test.

**Table 2:** Effect of MHB on plasma components in mice fed on HFD with or without MHB supplementation.

	Cont	MHB
NEFA (mEq/L)	0.58 ± 0.03	0.45 ± 0.02**
triacylglycerol (mM)	0.66 ± 0.04	0.68 ± 0.05
total cholesterol (mM)	4.70 ± 0.14	5.07 ± 0.11
glucose (mM)	8.67 ± 0.44	9.91 ± 0.54

Data are presented as means ± SEM. n = 12 mice/group.

**P < 0.01 (by unpaired Student’s *t*-test). NEFA, non-esterified fatty acid.

### MHB induced an increase in the mRNA levels of genes related to thermogenesis and fatty acid oxidation in BAT of mice

**Fig 2:**
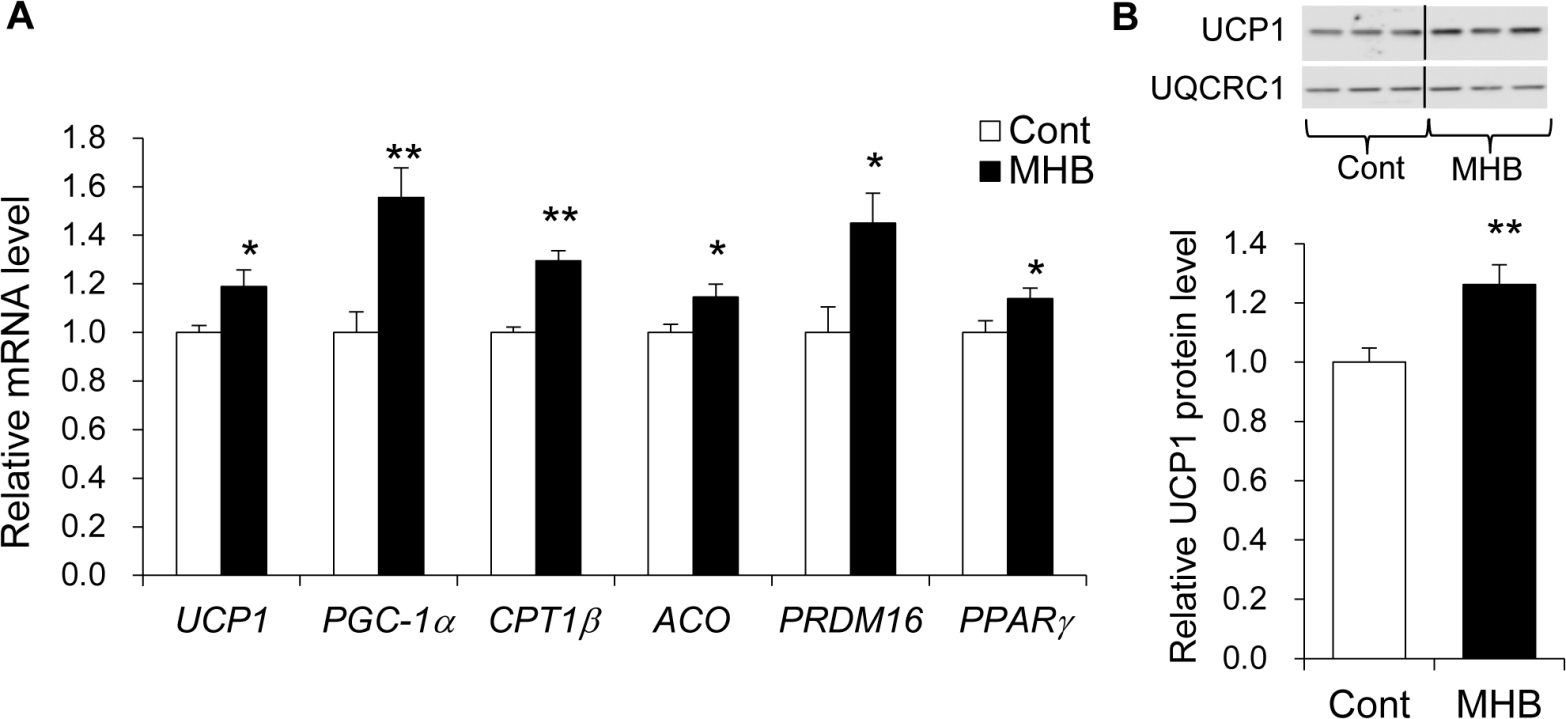
Measurement of mRNA expression levels and Immuno-blot analysis in the interscapular BAT of mice fed HFD supplemented with MHB. (A) mRNA expression levels in BAT. mRNA expression levels were normalized to the expression level of *GAPDH* as a reference. (B) Immuno-blot analysis of UCP1 in BAT mitochondria. Representative immuno-blots are shown on the histogram. Representative signals shown are from the same immuno-blot membrane. UCP1 protein level was normalized to the level of UQCRC1 protein as a reference. Data are presented as means ± SEM. n = 12 mice/group. *P < 0.05, **P < 0.01 (by unpaired Student’s *t*-test).

To elucidate the underlying mechanisms of the effects of MHB, quantitative RT-PCR was carried out using tissues from mice after 9 wk feeding of MHB. Supplementation with MHB significantly increased the mRNA levels of *PGC-1α*, *UCP1*, *CPT1β* and *ACO* genes by 1.6-fold, 1.2-fold, 1.3-fold, 1.2-fold in BAT, respectively (Fig 2A). The mRNA levels of *PRDM16* and *PPARγ* were also significantly increased by feeding MHB. There was also a concomitant increase in the UCP1 protein level in BAT mitochondria isolated from MHB fed mice (1.3-fold, *P* < 0.01) (Fig 2B). Although the mRNA levels of *FAS* in the liver and *ACC1* in the gastrocnemius muscle were significantly increased in the MHB-fed mice, no inclusive changes of gene expression were observed in the gastrocnemius muscle and liver between the groups (S4 Fig). These results indicate that MHB induces an increase in the mRNA levels of genes related to thermogenesis and fatty acid oxidation in BAT.

### Single oral administration of MHB led to elevated BAT-SNA in rats

**Fig 3:**
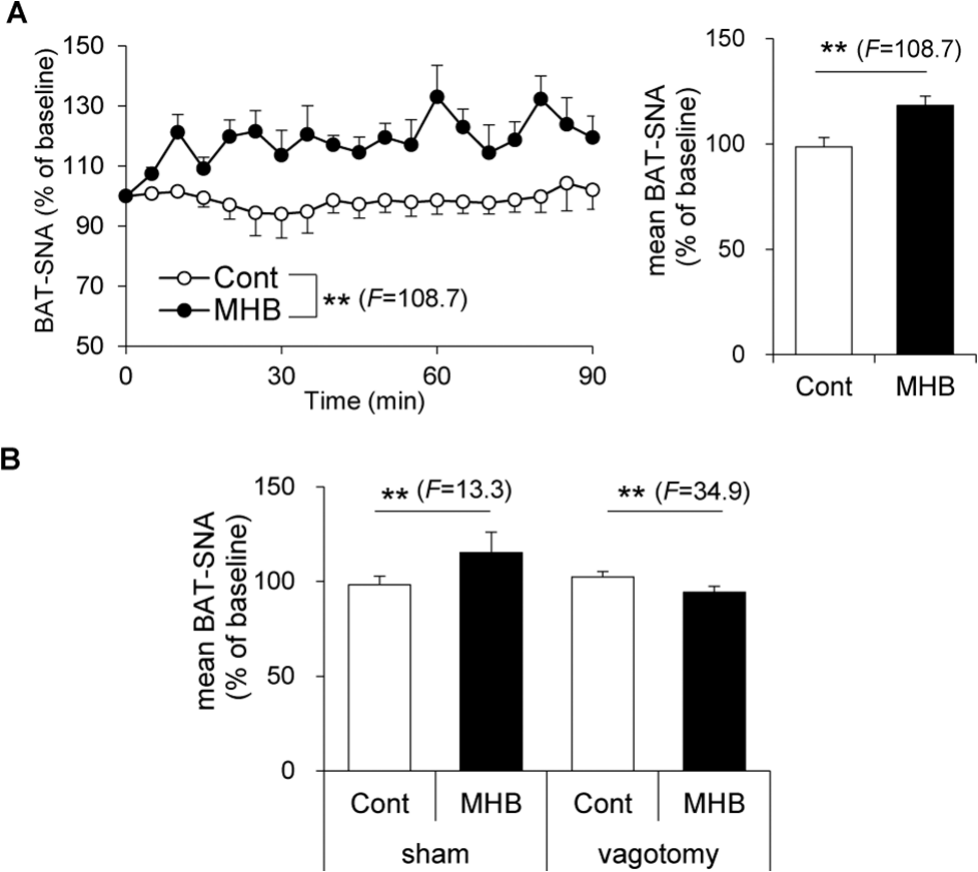
Single oral administration of MHB elevated BAT-SNA in rats. (A) Time course of BAT-SNA changes taken every 5 min and mean BAT-SNA over 0- to 90- min period. Urethane-anesthetized rats were administered 10 mg/kg of MHB. Data were calculated as a percentage of baseline, and as means ± SEM. n = 3 rats/group. **P < 0.01 (by ANOVA with repeated measures). (B) Effects of 2 mg/kg MHB on mean BAT-SNA over 0- to 90- min period in sham-operated or vagotomized rats. Data are presented as means ± SEM. n = 3 rats/group. **P < 0.01 (by ANOVA with repeated measures).

Because BAT is mainly innervated by sympathetic nerves [5], we orally administered MHB at a dose of 10 mg/kg and investigated changes in BAT-SNA in rats. MHB significantly elevated BAT-SNA compared to the control group (Fig 3A, *P* < 0.01). There was no significant statistical difference between the initial values of the two groups (Mann-Whitney *U* test). Subdiaphragmatic vagotomy completely abolished the observed effect of MHB (Fig 3B). There was no significant statistical difference between the initial values of the sham and vagotomized rats (Mann-Whitney *U* test). This finding suggests that vagal afferent nerves are involved in MHB induced elevation of BAT-SNA.

### Single oral administration of MHB increased the cAMP level and the temperature of BAT in rats

**Fig 4:**
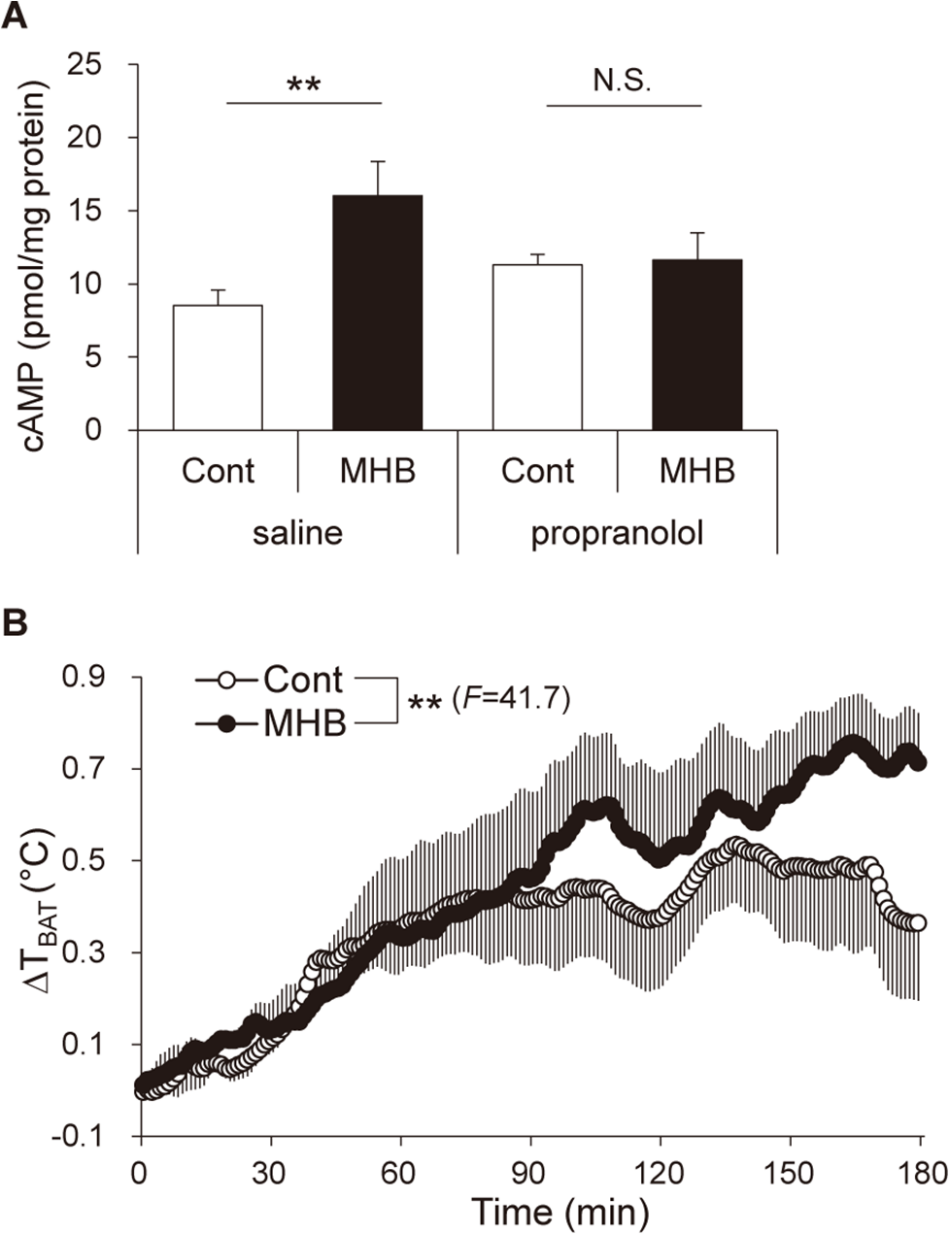
Single oral administration of MHB increased the level of cAMP in BAT and elevated its temperature in rats. (A) cAMP level in the lysates of BAT obtained from rats administered MHB pretreated with propranolol or saline. Thirty minutes after the pretreatment with 10 mg/kg propranolol or saline, rats were administered 10 mg/kg MHB and killed after 3 h. Data are presented as means ± SEM. n = 10 rats/group. **P < 0.01, N.S., not significant (by unpaired Student’s *t*-test). (B) Time course of BAT temperature changes (ΔT_BAT_) taken every 1 min relative to the baseline. The baseline was determined as the mean T_BAT_ over a 5-min period before the administration of MHB. Urethane-anesthetized rats were administered 10 mg/kg of MHB. Data are presented as means ± SEM. n = 4 rats/group. **P < 0.01 (by ANOVA with repeated measures).

In general, norepinephrine, which is secreted from the sympathetic nerve endings, induces cAMP accumulation *via* the β3-adrenergic signaling cascade in BAT [4,24]. To confirm the involvement of this mechanism, we measured the cAMP level in BAT 3 h after MHB administration at a dose of 10 mg/kg with or without pretreatment of β-adrenergic antagonist propranolol in rats. MHB induced an increase in the cAMP level (1.9-fold, *P* < 0.01), while pretreatment with propranolol completely suppressed cAMP induction by MHB (Fig 4A). To confirm whether elevation of BAT-SNA by MHB promotes thermogenesis in BAT, we measured the temperature of BAT (T_BAT_) in rats using a temperature transmitter. Single oral administration of MHB (10 mg/kg) significantly elevated T_BAT_ compared to that in the control group as shown in Fig 4B (*P* < 0.01). There was no significant statistical difference between the initial values of the two groups (Mann-Whitney *U* test).

## Discussion

Some of the beneficial effects associated with ingesting specific components of hops have been reported previously. These include the cancer chemopreventive activity of xanthohumol [25,26], estrogenic activity of 8-prenylnaringenin [25,27], cyclooxygenase-2 inhibition of humulone (an α-acid) [28], and prevention of diet-induced obesity of iso-α-acids [11]. Here, we show that ingestion of MHB, derived from hops, can induce thermogenesis in BAT and ameliorate diet-induced body fat accumulation. Taken together, these results provide scientific support for the empirical utilization of hops over many years in herbal medicines [29].

MHB did not induce gene expressions of *ACO* and *MCAD*, which are involved in β-oxidation and known to be induced by PPARα in the liver [30], or induce a suppressive effect on lipid absorption, while they are thought to be associated with the mechanism by which iso-α-acids prevent diet-induced obesity [11]. Intriguingly, these results indicate that the body fat accumulation-ameliorating mechanism of MHB could be different from that of iso-α-acids, despite the structural similarity of MHB components with iso-α-acids [14,15]. Thus, in order to reveal the differences between the mechanism of iso-α-acids and MHB, a more comprehensive investigation will be needed to identify all the active ingredients in MHB. Further structural information of multiple compounds found in MHB will reveal additional valuable data concerning the mechanism by which MHB reduces body fat accumulation.

Here, we found MHB supplementation induced a higher level of *PGC-1α* expression in BAT. PGC-1α is known to be a crucial regulator of gene expression related to thermogenesis and fatty acid oxidation such as *UCP1*, *ACO* and *CPT1β* genes [31,32]. Indeed, the expression of these genes in BAT was increased by MHB supplementation in the diet. By contrast, ingestion of MHB had no inclusive effect on the expression levels of genes involved in the beneficial effect of MHB on body fat accumulation in the gastrocnemius muscle and liver. Our results suggest that the effect of MHB is elicited, at least in part, by targeting BAT.

BAT thermogenesis is thought to be mediated by UCP1. Thus, control of UCP1 expression is considered to be an important factor for thermoregulation, energy expenditure regulation and maintenance of body weight [4,33]. Accordingly, activation of thermogenesis by increased expression of UCP1 in BAT could explain, at least in part, the body fat accumulation-ameliorating effect of MHB. The main energy source of thermogenesis is free fatty acids, which are first released from brown adipocytes and then from circulating fatty acids and lipoproteins [4,34]. MHB reduced the plasma NEFA level in mice fed HFD, suggesting that MHB may facilitate free fatty acid consumption in BAT by activation of thermogenesis.

In this study, single oral administration of MHB activated BAT-SNA in addition to elevating the level of cAMP and maintaining the temperature of BAT at a higher level than that of the control group in rats. Moreover, vagotomized rats completely lost the MHB induced activation of BAT-SNA, and the block on the β-adrenergic signaling cascade eliminated cAMP elevation brought on by MHB. These data suggest that induction of thermogenesis in BAT by MHB is mediated by the β-adrenergic signaling cascade *via* BAT-SNA activation, which follows the vagal afferent nerve activation. The mechanism of the vagal afferent nerve activation by MHB is unclear at the present time. One possibility is the involvement of taste receptors expressed in endocrine cells within the gut mucosa [35]. In the gut, taste receptors release hormones or neurotransmitters in response to basic tastants, which can communicate with the brain directly, *via* the bloodstream, or indirectly, *via* the vagal afferent nerves [35]. Because MHB activated BAT-SNA *via* vagal afferent nerves by oral administration into the gastric cavity, it is suggested that MHB may have the effect by modulating bitter taste receptors or other receptors in endocrine cells within the gut rather than those on the tongue. Intensive investigations will be needed to reveal the mechanism of the vagal afferent nerve activation by MHB.

In addition, it has been reported that chronic sympathetic stimulation, such as repeated administration of β3-adrenergic receptor agonists and repeated cold exposure, results in increased amounts of UCP1 protein and increased BAT [36]. MHB supplementation for 9 wk increased the level of UCP1 in BAT both at the mRNA and protein levels, suggesting that these effects may have been mediated by the chronic sympathetic stimulation *via* BAT-SNA. The cAMP signaling pathway has also been reported to lead to increased *PPARγ* gene expression in brown adipocytes [37]. MHB supplementation for 9 wk in mice significantly increased *PPARγ* gene expression level in BAT, suggesting that it is possible that MHB induces an increase in the *PPARγ* gene expression level *via* cAMP signaling pathway. It has been reported that the cAMP signaling and PPARγ signaling pathways synergistically increase the *UCP1* gene expression level [37]. Thus, it is possible that the PPARγ signaling pathway is also related to the induction of *UCP1* gene expression by MHB. It has been reported that acute activation of the β3-adrenergic signaling cascade induces thermogenesis by enhancing UCP1 activity *via* activation of lipolysis, while chronic activation of this cascade induces thermogenesis by increasing the UCP1 expression level as well as the biogenesis of mitochondria themselves [36]. Our findings, together with those of previous studies, suggest that continuous activation of BAT-SNA by MHB may induce thermogenesis by both an acute effect, which enhances UCP1 activity, and a chronic effect, which increases the UCP1 expression level. This enhanced thermogenesis induced by MHB may contribute to body fat reduction.

In our study, T_BAT_ changes were observed 90 min after a single oral administration of MHB, whereas continuous BAT-SNA elevation was detected after only 10 min of MHB administration. The reason for this apparent discrepancy is unclear at the present time. One possibility is that these temporal differences may reflect the time-lag of sequential physiological responses between BAT-SNA activation and T_BAT_ elevation. Alternatively, single oral administration of MHB may elevate T_BAT_ by distinct mechanisms other than those involving BAT-SNA. For example, MHB may contain direct activators of BAT thermogenesis, such as a β3-adrenergic agonist [38] and a TGR5 agonist [39], or affect the endocrine/paracrine activators, such as heart-derived natriuretic peptides (NP) [40] and fibroblast growth factor 21 (FGF21) [41]. More detailed investigations will be needed to fully understand the effect of MHB on BAT activation. Besides, it has been reported that UCP1-positive brown fat-like adipocytes, named ‘beige’ adipocytes, in WAT are induced under various physiological and pharmacological conditions by activating adipocyte receptors such as β3-adrenergic receptors and PPARγ [42]. The emergence of beige adipocytes in WAT is associated with protection against obesity and related disorders in rodent models [42]. In future studies, it should be explored whether MHB has an effect on inducing these beige adipocytes in WAT, not only on inducing thermogenesis in BAT.

In conclusion, our results reveal that single oral administration of MHB activates BAT-SNA, which induces thermogenesis in BAT of rats. In addition, administration of MHB-supplemented HFD to mice increases the expression level of UCP1 in BAT and ameliorates diet-induced body fat accumulation. Therefore, MHB could be a useful tool in providing functional foods and beverages to reduce diet-induced body fat accumulation and related metabolic disorders. However, additional studies will be needed to better understand the mechanism by which MHB ameliorates body fat accumulation.

## Supporting Information

S1 Fig HPLC analysis of MHB. (A) HPLC chromatogram of MHB detected at 270 nm. (B) HPLC chromatogram of ICE2 (standards of α- and β-acids) at 270 nm. (C) HPLC chromatogram of isomerized hop extract at 270 nm. (D) HPLC chromatogram of standard of tricyclooxyisohumulone A at 270 nm. (E) HPLC chromatogram of standard of tricyclooxyisohumulone B at 270 nm. Chemical structures are shown in S2 Fig.
(TIF)

S2 Fig Structure of α- and β-acids and their derivatives. α-acids: cohumulone (**1a**), *n*-humulone (**1b**), adhumulone (**1c**); β-acids: colupulone (**2a**), *n*-lupulone (**2b**), adlupulone (**2c**); *trans*-iso-α-acids: *trans*-isocohumulone (**3a**), *trans*-iso-*n*-humulone (**3b**), *trans*-isoadhumulone (**3c**); *cis*-iso-α-acids: *cis*-isocohumulone (**4a**), *cis*-iso-*n*-humulone (**4b**), *cis*-isoadhumulone (**4c**); tricyclooxyisohumulone A (**5**); tricyclooxyisohumulone B (**6**).
(TIF)

S3 Fig Food intake and correlation between total food intake and body weight gain in mice fed HFD supplemented with MHB. (A) Food intake of HFD-fed mice with or without MHB supplementation. Data are expressed per body weight, and as means ± SEM. n = 12 mice/group. No significant difference was observed by ANOVA with repeated measures. (B) Correlation between total food intake and body weight gain in mice fed on HFD with (●) and without (○) MHB supplementation. n = 12 mice/group. There was no significant correlation between total food intake and body weight gain (Pearson correlation coefficient *r* = 0.21, *p* = 0.32).
(TIF)

S4 Fig Measurement of mRNA expression levels in the liver and gastrocnemius muscle of mice fed HFD supplemented with MHB. (A) mRNA expression in the liver. (B) mRNA expression in the gastrocnemius muscle. The mRNA level was normalized to that of *GAPDH*. Data are presented as the relative level to the control group, and as means ± SEM. n = 12 mice/group. *P < 0.05 (by unpaired Student’s *t*-test).
(TIF)

S1 Table Primer sequences used for real-time PCR. (DOC)
