# Supplementary figures and images for: Matured Hop Bittering Components Induce Thermogenesis in Brown Adipose Tissue via Sympathetic Nerve Activity
Source: PLoS One. 2015 Jun 22;10(6):e0131042. doi: 10.1371/journal.pone.0131042 (PMC4476742; doi:10.1371/journal.pone.0131042)

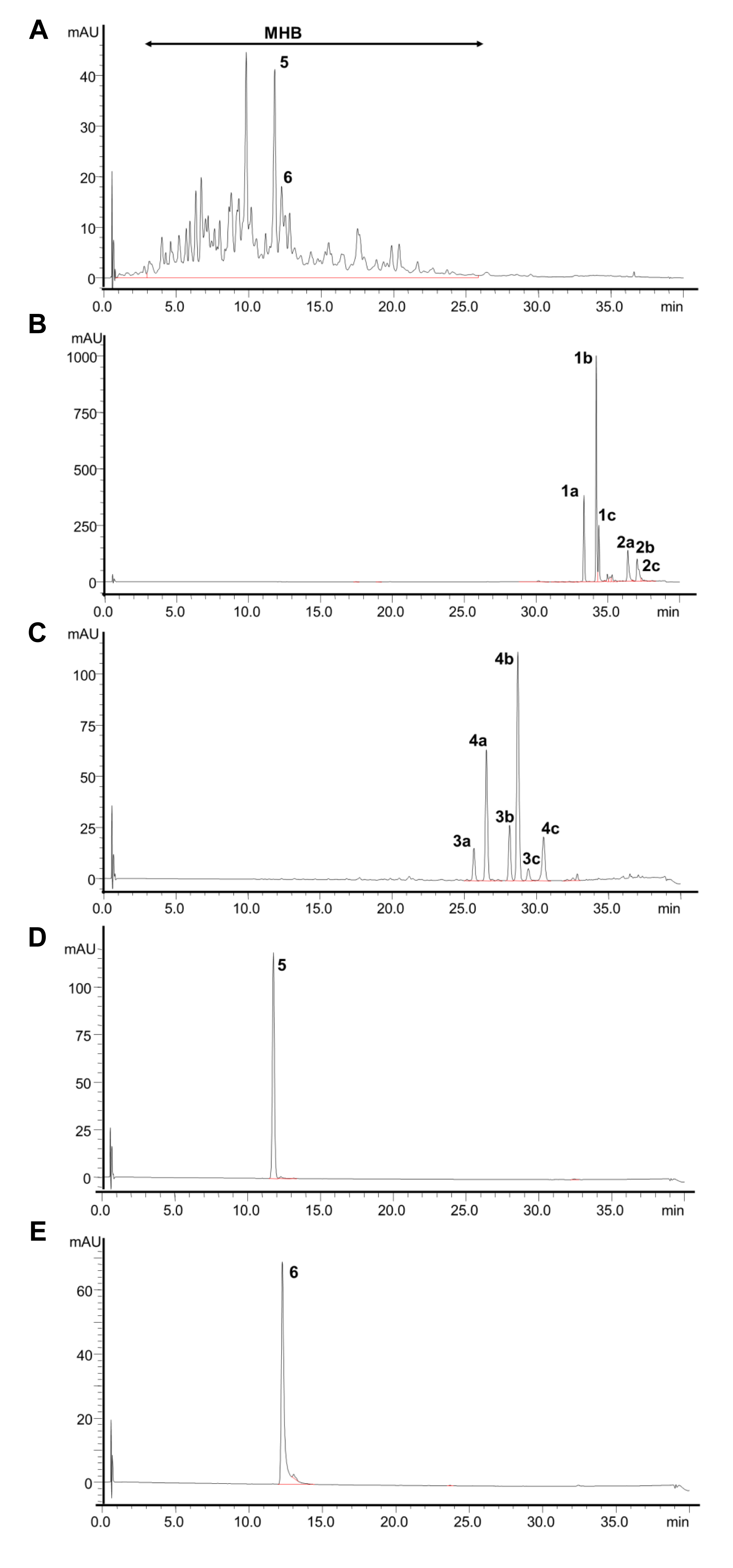

Supplement: S1 Fig — (A) HPLC chromatogram of MHB detected at 270 nm. (B) HPLC chromatogram of ICE2 (standards of α- and β-acids) at 270 nm. (C) HPLC chromatogram of isomerized hop extract at 270 nm. (D) HPLC chromatogram of standard of tricyclooxyisohumulone A at 270 nm. (E) HPLC chromatogram of standard of tricyclooxyisohumulone B at 270 nm. Chemical structures are shown in S2 Fig. (TIF) [file pone.0131042.s001.tif]

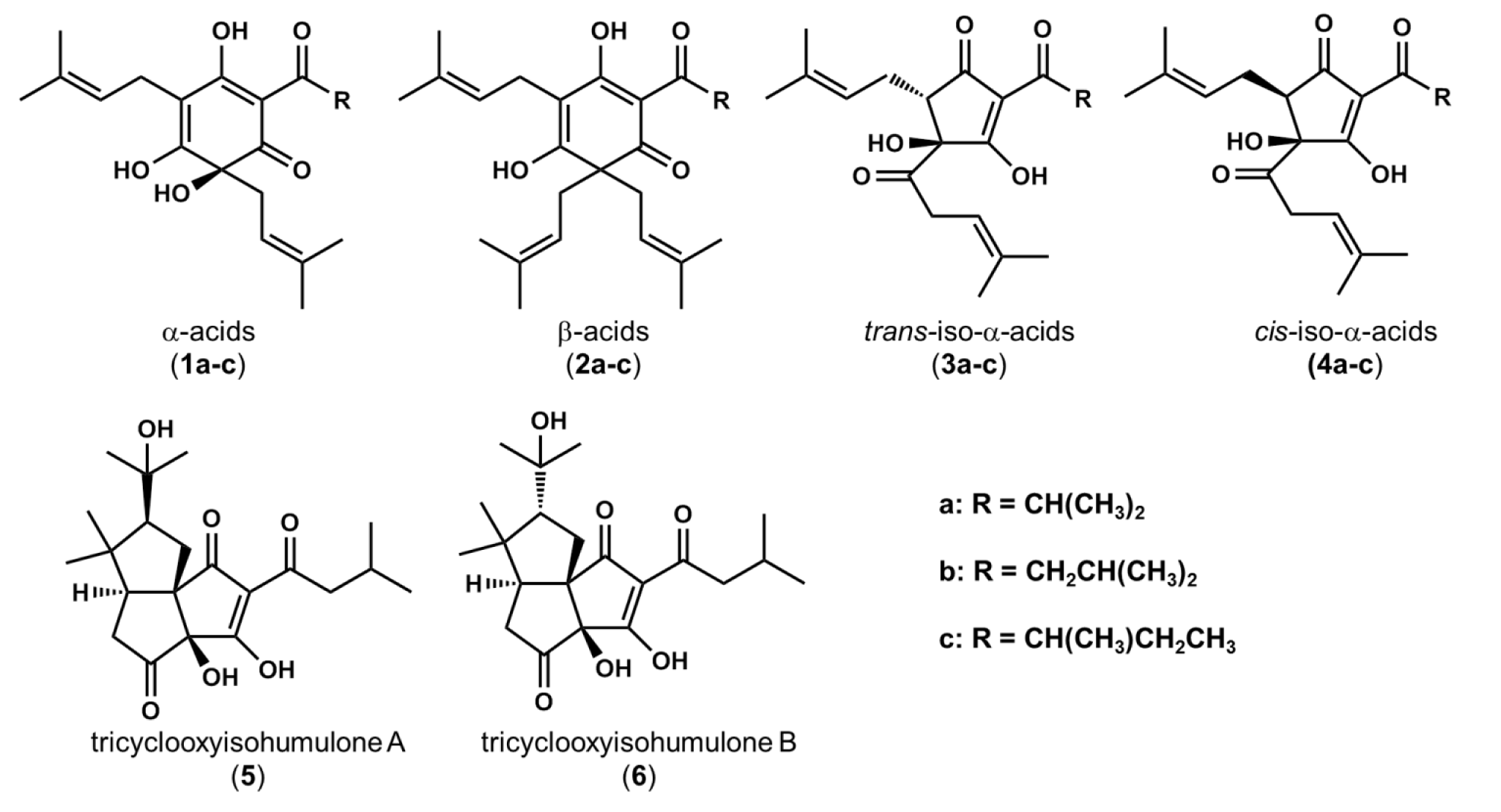

Supplement: S2 Fig — α-acids: cohumulone (1a), n-humulone (1b), adhumulone (1c); β-acids: colupulone (2a), n-lupulone (2b), adlupulone (2c); trans-iso-α-acids: trans-isocohumulone (3a), trans-iso-n-humulone (3b), trans-isoadhumulone (3c); cis-iso-α-acids: cis-isocohumulone (4a), cis-iso-n-humulone (4b), cis-isoadhumulone (4c); tricyclooxyisohumulone A (5); tricyclooxyisohumulone B (6). (TIF) [file pone.0131042.s002.tif]

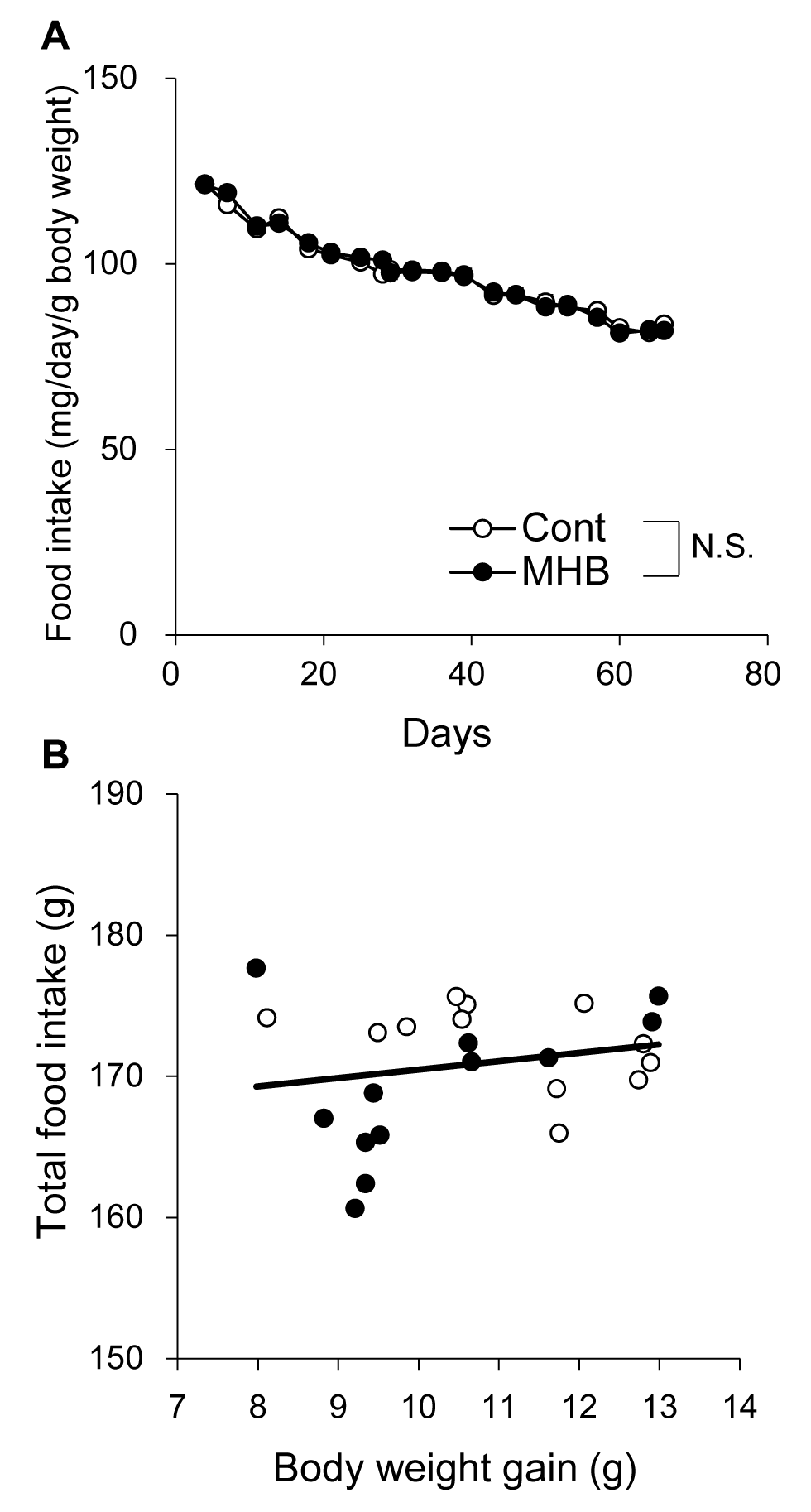

Supplement: S3 Fig — (A) Food intake of HFD-fed mice with or without MHB supplementation. Data are expressed per body weight, and as means ± SEM. n = 12 mice/group. No significant difference was observed by ANOVA with repeated measures. (B) Correlation between total food intake and body weight gain in mice fed on HFD with (●) and without (○) MHB supplementation. n = 12 mice/group. There was no significant correlation between total food intake and body weight gain (Pearson correlation coefficient r = 0.21, p = 0.32). (TIF) [file pone.0131042.s003.tif]

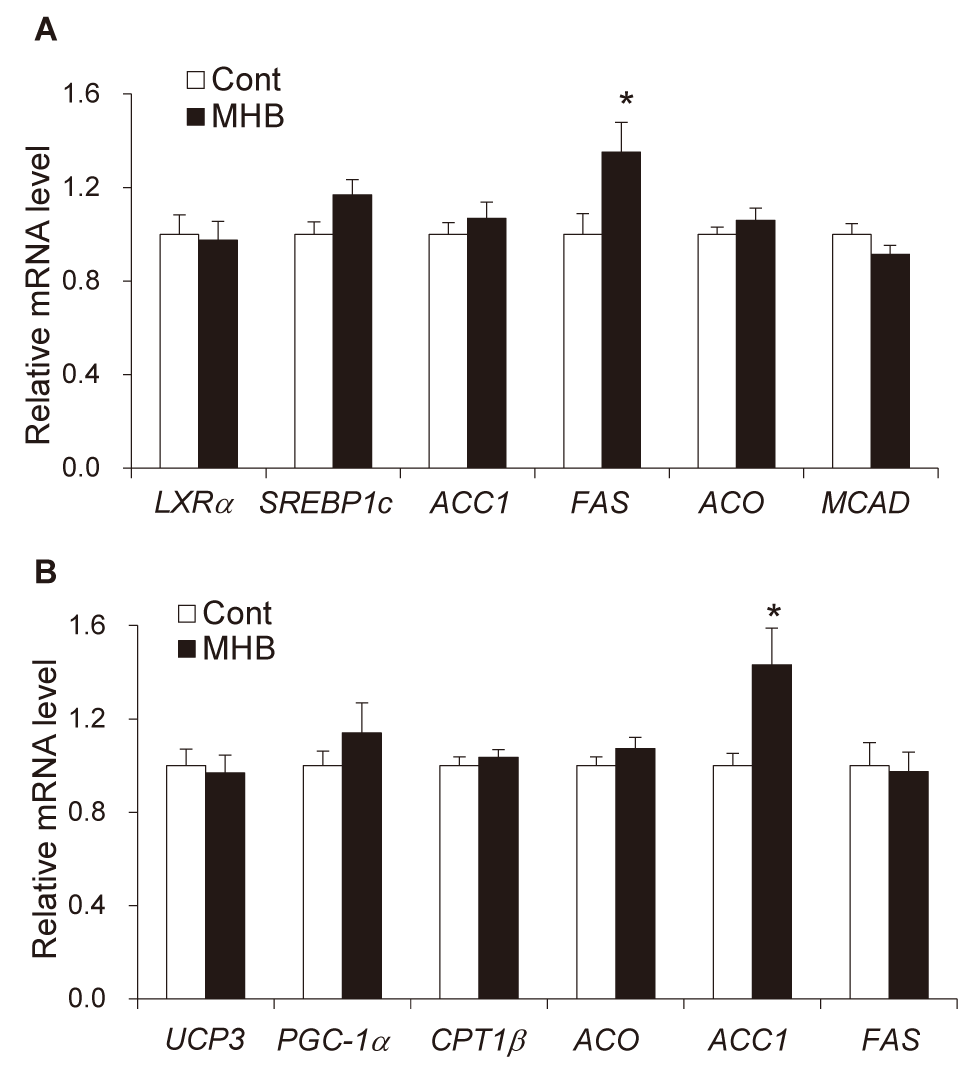

Supplement: S4 Fig — (A) mRNA expression in the liver. (B) mRNA expression in the gastrocnemius muscle. The mRNA level was normalized to that of GAPDH. Data are presented as the relative level to the control group, and as means ± SEM. n = 12 mice/group. *P < 0.05 (by unpaired Student’s t-test). (TIF) [file pone.0131042.s004.tif]
