# Supplementary material for: Matured Hop Bittering Components Induce Thermogenesis in Brown Adipose Tissue via Sympathetic Nerve Activity
Source: PLoS One. 2015 Jun 22;10(6):e0131042. doi: 10.1371/journal.pone.0131042 (PMC4476742; doi:10.1371/journal.pone.0131042)
Supplement: S1 Table — (DOC) [file pone.0131042.s005.doc]

**Table S1. Primer sequences used for real-time PCR.**

| Gene | Forward primer (5’-3’) | Reverse primer (5’-3’) |
| --- | --- | --- |
| *UCP1* | CAGAAGGATTGCCGAAACTG | ATGAACACTGCCACACCTCC |
| *PGC-1α* | TCCGAGCGGAGCTGAACAAGCA | AATAGGGCTGCGTGCCATCCCAAG |
| *CPT1β* | AACGTGCTGCTTTCTTTGTGACCC | AAACTCCCAGAGGTGCCCAATGAT |
| *ACO* | ATCTATGACCAGGTTCAGTCGGGG | CCACGCCACTTCCTTGCTCTTC |
| *PRDM16* | GCGAGCGGATGTTCCCCAACAA | TTCGAAGCGCTTGCCACTGTCG |
| *PPARγ* | TCTGCGAGCCCTGGCAAAGCAT | TGGCCACCTCTTTGCTCTGCTCCT |
| *LXRα* | AAAAGGGCCCAGCCCCCAAAATG | CCTGGCATTTCCGCCGCATGTA |
| *SREBP-1c* | TTTGCCTTCCAGCCCTCCACCA | TGGAAGTGTGCGGCCTGTGGAT |
| *ACC1* | TTGCGGGAATGTTCCCAGCCTGT | CCCGACGCATGGTTTTCACCAGA |
| *FAS* | TGCAGTGGGGTGCCATTGGTGA | TCCCCATGGGCCACAGCTTTCTTC |
| *MCAD* | AAATGCCTGTGATTCTTGCTGG | TTCCCCCGTTGGTTATCCAC |
| *UCP3* | GAAGGTCCGATTTCAAGCC | AAACAGGTGAGACTCCAGCAAC |
| *GAPDH* | TCTGCCGATGCCCCCATGTTTG | TGGGTGGCAGTGATGGCATGGA |
